# A platform approach as a plausible option for nonclinical safety assessment of adjuvanted vaccines

**DOI:** 10.1038/s41541-025-01245-3

**Published:** 2025-08-13

**Authors:** Eric Destexhe

**Affiliations:** https://ror.org/00n3pea85 grid.425090.a 0000 0004 0468 9597 GSK, Rue de l’Institut 89, 1330 Rixensart, Belgium

**Keywords:** Vaccines, Adjuvants

## Abstract

Vaccine platform technologies are reproducible, standardized manufacturing and development methods that may be leveraged to streamline regulatory approval of new vaccines. To evaluate the plausibility of a platform approach for the nonclinical safety assessment of adjuvanted recombinant-protein vaccine candidates, a comparative analysis was performed on repeat-dose toxicity data of four AS01 adjuvanted candidate vaccines from five rabbit studies. Groups of animals received AS01-adjuvanted vaccines, AS01 alone, or antigens alone on multiple occasions. All vaccines were well-tolerated, showing mainly transient signs of inflammation after the administrations, consistent across all vaccines and similar to AS01. The antigens alone induced only minimal signs of inflammation, indicating that AS01 mainly drives the innate immune response. These findings support the plausibility of a platform approach for the nonclinical safety assessment of adjuvanted vaccines, potentially reducing animal use and expediting first-in-human trials by streamlining or eliminating some nonclinical studies.

## Introduction

Platform technologies form the basis upon which other applications, processes or technologies are developed^1^. The Food and Drug Administration (FDA) defines in a draft guidance a platform technology as a well-characterized, reproducible technology integral to a drug’s or biologic’s structure or function, used in multiple products, and facilitating standardized manufacturing or development^2^. The FDA’s Platform Technology Designation (PTD) program is developing the process for leveraging platform information in regulatory submissions, streamlining drug, biologic, and vaccine development, manufacturing, and review^2^. Leveraging nonclinical safety data from prior products can potentially reduce the need for product-specific repeated-dose toxicity (RDT) studies^1–3^.

Vaccine RDT studies are typically performed before first-in-human clinical testing and are needed for nonclinical safety assessment^4^. These studies involve measurements in animals of numerous endpoints at multiple time points. Before 1997, RDT studies were limited or nonexistent, often lacking microscopic tissue evaluation^5^. Many older vaccines (e.g., killed, attenuated, conjugate) underwent limited nonclinical testing^5–7^. On the contrary, subsequent RDT studies of new vaccine candidates, including those sharing a technology, such as an adjuvant, have generated extensive data. Analyzing such RDT data from adjuvanted vaccines may reveal adjuvant-specific changes that are dependent on the adjuvant and independent of the antigen. Such information could support the development of new vaccines with different antigens using the same adjuvant based on prior RDT data, aligning with the FDA’s PTD program^2,8^.

Adjuvants are designed to enhance or modulate immune responses to antigens, thereby improving clinical effectiveness of vaccines^9^. Among the adjuvants, Adjuvant Systems (AS) contain multiple immunomodulators that interact with each other in complex ways and selected for a specific modelling of the immune response and the target population. The Adjuvant System(AS)01 (GSK)^10,11^ is a component of several vaccine candidates and of the licensed vaccines *Shingrix*, *Mosquirix*, and *Arexvy* (all GSK). AS01 comprises 3-*O*-desacyl-4’-monophosphoryl lipid A (MPL), QS-21 (*Quillaja saponaria* Molina, fraction 21) and liposomes. MPL, a detoxified *Salmonella minnesota* lipopolysaccharide derivative, is a toll-like receptor 4 (TLR4) ligand, while QS-21 is a triterpene glycoside from *Q. saponaria* bark.

Recombinant proteins as vaccine antigens are generally well tolerated and can be genetically modified to eliminate any intrinsic toxicity. By themselves recombinant proteins are poorly immunogenic, and adjuvants can be used to enhance their immunogenicity, which is usually associated with an increased reactogenicity^10^.

Since the manufacturing of adjuvants and recombinant proteins are based on well-established and robust processes, a platform approach is a plausible option for the nonclinical safety evaluation of adjuvanted vaccines. To assess this hypothesis, data from five RDT studies conducted with four AS01-adjuvanted, recombinant-protein, vaccine candidates were retrospectively reviewed, comparing the AS01-adjuvanted vaccines with each other, AS01 alone, and/or antigens alone.

## Results

### Study design

**Fig. 1:**
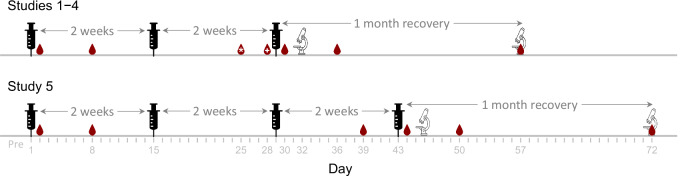
Study design of the RDT studies conducted in rabbits. Syringes indicate the days of intramuscular injections, blood drops signify the days of sampling for measuring clinical pathology endpoints, and microscopes mark the days of euthanasia for organ weight and macroscopic and microscopic assessments. Pre-dose samples are not shown. Serology sampling days are shown in Table 1. In the blood drops, the asterisk and the plus signs indicate that blood samples were taken only in Study 4 and Study 3, respectively.

Five RDT studies were reviewed, which were conducted in OECD member countries with four AS01-adjuvanted vaccine candidates targeting two viral and one bacterial pathogens, and comprising four distinct recombinant-protein antigens, one of which was assessed at two dose levels in separate studies. The antigens were produced in Chinese hamster ovary (CHO) cells or *Escherichia coli*. The studies were conducted in New-Zealand White rabbits at three different contract research organizations over a span of seven years. The intended human vaccine doses (0.5 mL per dose) were administered to the rabbits via three or four intramuscular injections spaced two weeks apart, and both local reactions and systemic effects were assessed (Supplementary Table 1), as well as their persistence, delayed onset, and reversibility after a one-month recovery following the final injection (Fig. 1, Table 1, and Supplementary Table 2). In addition to comparing the local reactogenicity and systemic toxicity of four AS01-adjuvanted vaccines with each other, the local reactogenicity and systemic toxicity of one AS01-adjuvanted vaccine was compared with AS01 alone, and three AS01-adjuvanted vaccines were compared with their respective antigens alone.

**Table 1:** RDT studies of AS01-adjuvanted vaccines conducted in rabbits and retrospectively reviewed

Study	Year of conduct	Place of study conduct	Antigen dose (µg)	Expression system	No. of IM doses	Dosing days	Clincial pathology sampling days	Serology sampling days
1^a^	2019	Covance (Greenfield, IN, USA)	Ag-A2^b^ (240)	CHO	3	1, 15, 29	Pre^c^, 2, 8, 30, 36, 57	Pre, 32, 57
2^a^	2018	Covance (Greenfield, IN, USA)	Ag-A1 (120)	CHO	3	1, 15, 29	Pre, 2, 8, 30, 36, 57	Pre, 32, 57
3^a^	2017	CiToxLAB (France)	Ag-B (120)	CHO	3	1, 15, 29	Pre, 2, 8, 28, 30, 36, 57	Pre, 32^f^, 57
4^a^	2012	TNO Triskelion (The Netherlands)	Ag-C (60)	*E. coli*	3	1, 15, 29	Pre, 2, 8, 25, 30, 36, 57	Pre, 32, 57
5^d^	2012	TNO Triskelion (The Netherlands)	Ag-D (62.5)	CHO	4	1, 15^e^, 29, 43	Pre, 2, 8, 39, 44, 50, 72	Pre, 44, 72

^a^Included an antigen alone group, as previously reported^17^.

^b^Ag-A2 contains two-fold more Ag-A than Ag-A1.

^c^Pre = Pre-dose samples.

^d^Included an AS01 alone group, as previously reported^18^.

^e^Males were injected on day 16 but for simplicity the day of injection for males and females is shown as Day 15 here and in the figures.

^f^Except the five principal females from group 3 that were sampled on Day 46.

### Comparisons of four vaccine candidates

#### In-life endpoints

Across all studies, no significant effects were observed on mortality, clinical findings, local reactions, body weight, food intake, ophthalmological assessments, or body temperature. However, minor body weight loss was observed following the second and/or third injections of the Ag-B/AS01 vaccine, which quickly recovered and was therefore considered non-adverse.

#### Immunogenicity

**Fig. 2:**
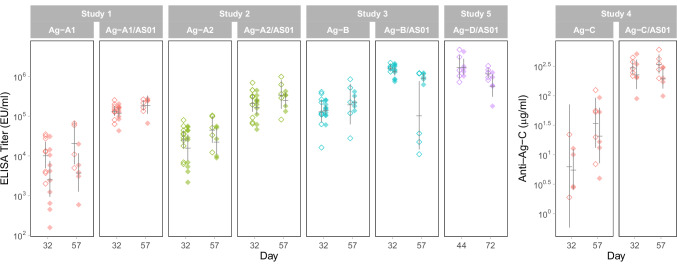
Vaccine uptake and adjuvant effect on immunogenicity. Diamonds represent titers per animal (open, females; closed, males). Crossbars indicate the geometric mean titers for the groups, with vertical lines representing the 95% confidence intervals for the geometric means.

The ability of the vaccine candidates and their antigens alone to generate antibody responses was verified, confirming the uptake of the vaccines (Fig. 2).

#### Clinical pathology

**Fig. 3:**
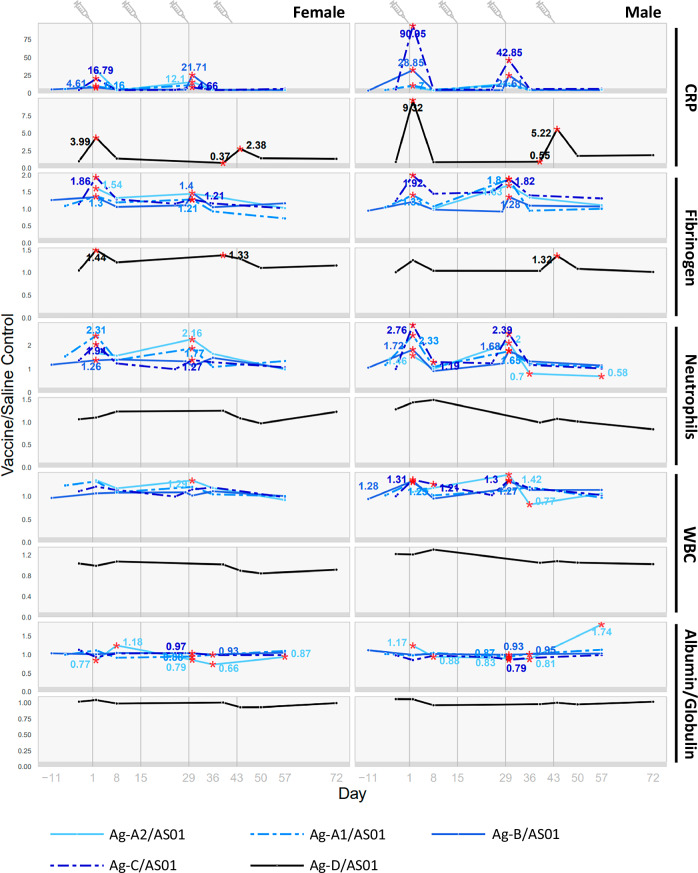
Changes in clinical pathology parameters (hematology, clinical chemistry, and coagulation) following administration of AS01-adjuvanted vaccine candidates to rabbits. The ratio of the means of the vaccine group to the saline-control group within each individual study are plotted. A red asterisk indicates that the mean of the vaccinated group differed significantly from the mean of the saline control group in the study (two-sided tests, *p* < 0.05). For points with a red asterisk, the values of the ratio of the means of the vaccine group to the saline-control group (fold-change) are shown as numbers next to the asterisks. Statistics were not calculated if one of the groups was above or below the limit of quantification. Pre-treatment time points are shown only if the groups to be treated with the test item or saline were sampled on the same day. The endpoints selected for graphing showed significant vaccine-related changes for four of five of the vaccine candidates.

Among the five studies, the significant changes in clinical pathology parameters are similar: neutrophil counts, white blood cell (WBC) counts, fibrinogen and C-reactive protein (CRP) levels increased, whereas the albumin-to-globulin ratio decreased (Fig. 3). By the end of the 1-month recovery period, most vaccine-related changes had partially or fully returned to control levels, except for Ag-A2/AS01, where the albumin-to-globulin ratio remained lower than the saline control in females. These changes in clinical pathology parameters indicate that the vaccines elicited a transient inflammatory reaction after the injections and the development of an immune response that is expected from an intramuscular administration of an immunogenic vaccine to rabbits^12^.

While the clinical pathology changes presented above were observed in at least four studies (i.e., there were no statistical changes observed for Ag-D/AS01 regarding neutrophil counts, WBC counts, and albumin-to-globulin ratio), other changes occurring after the administration of the vaccines are presented in Supplementary Table 3. These additional findings were not considered toxicologically meaningful due to the minimal magnitude of the changes, inconsistent direction between animals, and general overlap with control values.

#### Postmortem examinations

Postmortem macroscopic and microscopic examinations revealed an inflammatory reaction at the injection site (mixed cell infiltration in the muscle and subcutis) accompanied by myofiber degeneration/necrosis, and immune stimulation in draining lymph nodes and/or spleen for all AS01-adjuvanted vaccines (Table 2). By the end of the one-month recovery period after the last vaccination, injection-site reactions and spleen immune stimulation were almost fully reversible (Table 3), while evidence of immune stimulation was still present in the draining lymph nodes.

**Table 2:** AS01-adjuvanted vaccines–related injection site reaction and lymphoid organ immune stimulation after administration of AS01-adjuvanted vaccines to rabbits at terminal euthanasia

Study	Vaccine candidate	Injection-site inflammation	Draining LN weight increase	Draining LN enlargement	Draining LN immune stimulation	Spleen immune stimulation
1	Ag-A2	✓	✓	✓	✓	✓
2	Ag-A1	✓	✓	✓	✓	–
3	Ag-B	✓	✓	–	✓	✓
4	Ag-C	✓	–*	–	✓	✓
5	Ag-D	✓	✓*	–	✓	–

*Only the popliteal lymph nodes were weighed.

✓ Observed.

– Not observed.

**Table 3:** Evolution of injection site reactions and lymphoid organ immune stimulation after administration of AS01-adjuvanted vaccines to rabbits

ENDPOINTS EVALUATED	TERMINAL EUTHANASIA	RECOVERY EUTHANASIA
MICROSCOPY, INJECTION SITE	Minimal to moderate mixed inflammatory cells infiltrate extending into the subcutis Minimal to slight myofiber degeneration/necrosis	Almost fully reversible
ORGAN WEIGHTS	Increased iliac and popliteal lymph nodes	Partially reversible
MACROSCOPY	Enlargement of the popliteal lymph node	Fully reversible
MICROSCOPY, ILIAC AND POPLITEAL LYMPH NODES	Increased number of lymphocytes and neutrophils	Fully reversible

### Contribution of AS01 to changes induced by AS01-adjuvanted vaccines

Since AS01 was developed as a new adjuvant, a group administered AS01 alone was included in the RTD study conducted with Ag-D/AS01^13^. The AS01 group was compared with the Ag-D/AS01 group to assess the contribution of AS01 to the changes elicited by Ag-D/AS01.

#### In-life endpoints

There were no findings in either group, except that an immune response was directed against the antigen only in the Ag-D/AS01 group (Table 4).

**Table 4:** Changes elicited by AS01 and Ag-D/AS01 after intramuscular administration to rabbits

Outcome of the evaluated endpoints	AS01	Ag-D/AS01
No mortality	✓*	✓
No local reactions	✓	✓
No clinical observations	✓	✓
No changes in body weight & food consumption	✓	✓
No changes in body temperature	✓	✓
No ophthalmology findings	✓	✓
Immune response	–	✓

*One female on day 16 showed an umbilical hernia not affecting general health until death on day 37 due to partial entrapment of the jejunum in the umbilical hernia, resulting in jejunum necrosis and fatal toxemia that was unrelated to the treatment.

✓ Observed.

– Not observed.

**Fig. 4:**
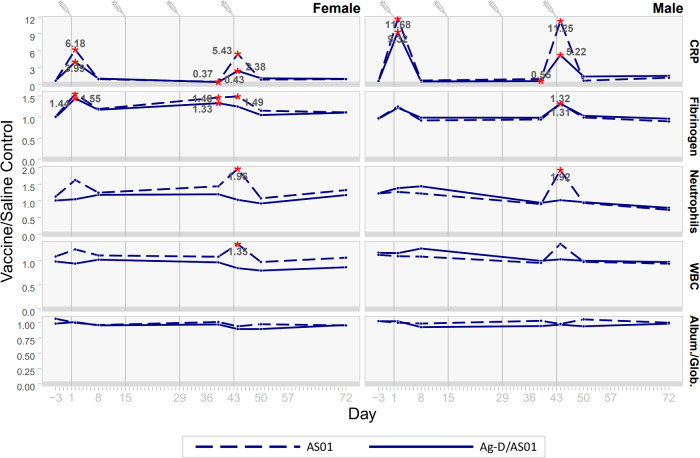
Changes in clinical pathology parameters (hematology, clinical chemistry, and coagulation) following administration of AS01 or Ag-D/AS01 to rabbits. A red asterisk indicates a significant difference between the AS01 group or the Ag-D/AS01 group and the saline-control group mean (two-sided tests, *p* < 0.05). Statistics were not calculated if one of the groups was above or below the limit of quantification. The gray numbers indicate the absolute fold-change compared with saline controls.

Changes observed in the Ag-D/AS01 and AS01 groups were similar for CRP and fibrinogen, while only the AS01 group showed increases in neutrophil and WBC counts (Fig. 4).

#### Postmortem examinations

Inflammation (mixed inflammatory cell infiltration) at the injection site was observed in the AS01 and Ag-D/AS01 groups, accompanied by myofiber degeneration in few animals, including one control. However, increased weight and immune stimulation of draining lymph nodes were only observed in the Ag-D/AS01 group (Table 5).

**Table 5:** Morphological changes following administration of AS01 or Ag-D/AS01 to rabbits

Endpoints evaluated	AS01	Ag-D/AS01
Inflammation at the injection site	✓	✓
Increased draining lymph nodes (LN) weights	–	✓*
Immune stimulation in the draining LN, heterophils	–	✓
Enlargement of draining LN	–	–
Immune stimulation in the spleen	–	–

*Only the popliteal lymph nodes were weighed.

✓ Observed.

– Not observed.

### Contribution of antigens to changes induced by AS01-adjuvanted vaccines

Of the five studies, four included a group that received the antigens alone in saline. These groups were compared with their respective AS01-adjuvanted groups to assess the contribution of the antigens to the vaccine-elicited changes.

#### In-life endpoints

**Fig. 5:**
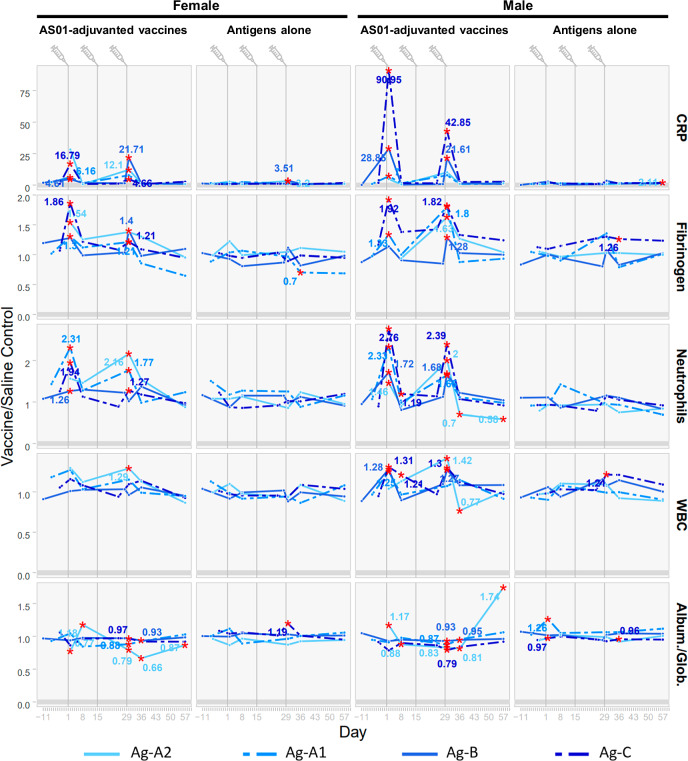
Changes in clinical pathology parameters (hematology, clinical chemistry, and coagulation) following administration of AS01-adjuvanted vaccines or antigens alone to rabbits. Red asterisks indicate that the mean of the test-article group differed significantly from the mean of the saline-control group in the original study (two-sided tests, *p* < 0.05). Statistics were not calculated if one of the groups was above or below the limit of quantification. The blue numbers indicate the fold-change versus saline controls. Pre-treatment time points are shown only if the groups to be treated with the test item or saline were sampled on the same day.

None of the antigens alone elicited changes in clinical condition, body weight, food consumption, dermal observations, or body temperature. However, after the third injection of Ag-B antigen, administered at the same dosage as in the vaccine, males and females experienced transient and minor weight loss, which was considered non-adverse due to its limited severity and lack of impact on the animals’ health and well-being. The antigens alone caused only minor changes in hematology, clinical chemistry and coagulation parameters, which were not considered toxicologically relevant due to their low magnitude (Fig. 5). However, females administered Ag-A2 experienced minimal increases in fibrinogen and CRP levels on the day after the first injection, suggesting that higher antigen amounts can elicit more pronounced changes.

On the contrary, the magnitude of the changes for the affected clinical pathology parameters was significantly higher in animals that received the adjuvanted vaccines in comparison to those that received the antigens only.

#### Postmortem examinations

Postmortem macroscopic and microscopic examinations of the animals that received the antigens alone revealed inflammatory reactions at the injection site which were of lower severity and/or incidence compared with those observed in the animals that received the respective AS01-adjuvanted vaccines, and an immune stimulation of the draining lymph nodes and spleen was also observed. By the end of the one-month recovery period following the last vaccination with the antigens alone, the injection-site reactions and immune stimulation were partially or completely reversible.

## Discussion

This review of RDT data encompassed five studies, conducted over a seven-year period (2012–2019) at three contract research organizations. These studies assessed four AS01-adjuvanted vaccine candidates targeting two viral and one bacterial pathogens with distinct recombinant-protein antigens, including one antigen evaluated at two dose levels. Three of the antigens (Ag-A, Ag-B, and Ag-D) were produced in CHO cells and one (Ag-C) in *E. coli*.

Recombinant-protein antigens present by themselves minimal risk for direct toxicity, as they are administered infrequently and in small amounts^5,14^. Although molecular mimicry can potentially lead to adverse events due to induction of autoimmune phenomena, no animal models currently exist to screen for the induction of autoimmunity^15^. On the contrary, toxicity studies are designed to evaluate changes in parameters linked to the innate and adaptative immune responses.

Despite variations in antigen identity, quantity, and manufacturing, all the AS01-adjuvanted vaccines were well-tolerated both locally and systemically, eliciting mainly transient inflammatory reactions of similar nature and intensity, as expected due to the mode of action of adjuvanted vaccine when administered intramuscularly.

AS01 alone elicited local and systemic reactions similar to those induced by the adjuvanted vaccine (Ag-D/AS01), although the adjuvanted vaccine also stimulated an adaptive immune response, evidenced by increased weights and immune activation of the draining lymph nodes, and the development of specific antibody responses, also reflected by the decreased albumin-to-globulin ratio.

The similarity in the nature of the findings, reflecting an innate immune response, induced by AS01 and AS01-adjuvanted vaccines suggests they were primarily driven by the adjuvant rather than the antigen. This is further supported by the almost absence of changes in clinical pathology parameters induced by the antigens alone, while signs of adaptive immunity were observed for both adjuvanted or unadjuvanted vaccines, although the magnitude of the response was more pronounced for adjuvanted vaccines. Based on these observations, it can be concluded that the adjuvant is the main contributor to the reactogenicity observed following the administration of adjuvanted vaccines.

Our results are consistent with previous studies comparing adjuvants alone with their respective adjuvanted vaccines, which show that the primary driver of reactogenicity is the adjuvant (12 studies)^12^. Additionally, our findings align with previous research comparing antigens alone to their respective adjuvanted vaccines (five studies), which also found that adjuvants are the major contributors to the reactogenicity^12^.

Collectively, these data support the plausibility of a platform approach for toxicity assessment of adjuvanted vaccines, such as described in the World Health Organization (WHO) and FDA regulatory guidance on platform-based vaccines, including COVID-19 mRNA vaccines^1,16^ and Ebola vaccines^3^. These guidelines suggest that nonclinical safety studies, including RDT studies, may be abbreviated or even omitted when sufficient platform safety data exists. Further endorsement of the platform approach to nonclinical safety studies via regulatory guidance is expected to reduce unnecessary animal use and facilitate the transition to first-in-human clinical trials without compromising the safety of new adjuvanted vaccines, especially when the only new component is the antigen from a well-characterized manufacturing process. This platform approach might also extend to developmental and reproductive toxicology (DART) studies when the defined conditions are met.

In summary, the consistent nature and severity of the transient inflammatory reactions, observed irrespectively of the antigen, its dose, or its manufacturing process, indicate that the adjuvant, rather than the antigen, was the predominant driver of the innate immune response associated with reactogenicity. Additionally, comparisons between antigens alone, AS01 alone and AS01-adjuvanted vaccines confirmed the major role of the adjuvant in eliciting the observed transient inflammatory changes following the vaccine administrations. These findings suggest that the toxicity evaluation of adjuvanted vaccines utilizing recombinant proteins and already characterized adjuvant can be effectively conducted through a platform-based approach, thereby reducing unnecessary animal testing while still ensuring the safety of patients in early clinical trials.

## Methods

### Study ethics and regulatory principles

The studies were conducted in accordance with the Organisation for Economic Cooperation and Development (OECD) Principles of Good Laboratory Practice and in compliance with all guidelines applicable at the time of study conduct for the evaluation of vaccines: CPMP Note for Guidance on Preclinical Pharmacological and Toxicological Testing of Vaccines (CPMP/SWP/465/95); WHO Guidelines on Nonclinical Evaluation of Vaccines (WHO Technical Report Series, No. 927, Annex 1, 2005); Guideline on Adjuvants In Vaccines For Human Use (Chmp/Veg/134716/2004); WHO Guidelines On The Nonclinical Evaluation Of Vaccine Adjuvants And Adjuvanted Vaccines (WHO Expert Committee On Biological Standardization, Sixty-Fourth Meeting, Who, 21-25 October 2013); Note For Guidance On Non-Clinical Local Tolerance Testing Of Medicinal Products (CPMP/SWP/2145/00). For studies 1 and 2, conducted at Covance, Greenfield, IN, USA, all procedures in the Protocol were in compliance with applicable animal welfare acts and were approved by the local Institutional Animal Care and Use Committee (IACUC). For study 3, CiToxLAB France Ethical Committee (CEC) reviews all the study plans to assess compliance with the corresponding authorized “project” as defined in the Directive 2010/63/EU. For studies 4 and 5 conducted at TNO Triskelion, Zeist, the Netherlands, the welfare of the animals was maintained in accordance with the general principles governing the use of animals in experiments of the European Communities (Directive 2010/63/EEC) and Dutch legislation (The Experiments on Animals Act, 1997). This included approval of the study by TNO’s ethical review committee.

GSK is committed to the replacement, reduction, and refinement of animal studies (3Rs), whenever possible. The studies were conducted in accordance with the GSK Policy on the Care, Welfare and Treatment of Laboratory Animals. The welfare of the animals was maintained in accordance with the general principles governing the use of animals in experiments, and for studies conducted in Europe, with the European Communities (Directive 86/609/EEC) and national legislations. Ethical reviews were done by an Ethical Committee or IACUC prior to experimentation.

### Animals and husbandry

New-Zealand White albino rabbits, specific pathogen-free bred, were supplied by Covance Research Products, Inc. (Denver, Pennsylvania, USA) or Centre Lago (Vonnas, France). They were identified using implantable microchips, cage cards, or ear tattoo codes and were individually housed in stainless steel cages, Noryl cages, or cages fitted with a perforated floor and a closed-bottom polypropylene box with bedding placed on the perforated floor, depending on the study. The females and males weighed between 1987–3870 g and 1832–3700 g, respectively, and were acclimated to the test facility housing conditions for 10 to 17 days prior to the first day of dosing, at which time they were between 12 and 16 weeks old. They were offered ad libitum Certified Rabbit Diet #2030 C, Breeding Pelleted Maintenance “type 110 C” diet, or Standard Laboratory Rabbit Diet (StanRab (P) SQC), depending on the study. Water was provided ad libitum. Environmental controls were set to maintain a temperature range of 16 to 22 °C, a relative humidity range of 30 to 70%, and a 12-hour light/12-hour dark cycle. For all studies at necropsy, the animals were deeply anesthetized by an intravenous injection of sodium pentobarbital and euthanized by exsanguination.

### Test and control articles

Formulations were prepared on each day of dosing. Ready-to-reconstitute vials containing lyophilized antigens were re-suspended in saline (0.9% Sodium Chloride for Injection, USP [sterile saline]) or AS01_B_ (50 μg QS21 and 50 μg MPL per 0.5 mL in a liposome-based formulation, used in all described experiments and denominated as AS01 throughout the text) and administered within 4–6 h of reconstitution. Dose formulations were held at ambient temperature prior to dosing. Syringes were filled to provide a 0.5 mL dose, equivalent to the intended human dose. QS21 has been licensed by GSK from Antigenics LLC, a wholly owned subsidiary of Agenus Inc., a Delaware, USA corporation.

### Study groups

A standard study design for RDT studies was used, similar to previous study^17,18^, as shown in Table 6.

**Table 6:** Experimental design for RDT Main and Recovery studies

Study	Antigen	AS01_B_	Injection^a^ Days	Main Study		Recovery Study	
				No. of Males	No. of Females	No. of Males	No. of Females
1	Saline	–	1, 15, 29	5	5	5	5
1	Ag-A2^b^	–	1, 15, 29	5	5	5	5
1	Ag-A2^b^	+	1, 15, 29	5	5	5	5
2	Saline	–	1, 15, 29	5	5	5	5
2	Ag-A1	–	1, 15, 29	5	5	5	5
2	Ag-A1	+	1, 15, 29	5	5	5	5
3	Saline	–	1, 15, 29	5	5	5	5
3	Ag-B	–	1, 15, 29	5	5	5	5
3	Ag-B	+	1, 15, 29	5	5	5	5
4	Saline	–	1, 15, 29	5	5	5	5
4	Ag-C	–	1, 15, 29	5	5	5	5
4	Ag-C	+	1, 15, 29	5	5	5	5
5	Saline	–	1, 15^c^, 29, 43	5	5	5	5
5	Ag-D	+	1, 15, 29, 43	5	5	5	5
5	–	+	1, 15, 29, 43	5	5	5	5

^a^0.5 mL per injection.

^b^Ag-A2 contains two-fold more Ag-A than Ag-A1.

^c^Males were injected on day 16 but for simplicity the day of injection for males and females is shown as Day 15 here and in the figures.

### Endpoints evaluated

The endpoints evaluated in this study are shown in Table 7. The individual tests and the individual tissues for gross pathological and/or microscopic examination and weighing are listed in Supplementary Table 1 and Supplementary Table 2, respectively.

**Table 7:** RDT study design

Endpoints evaluated	Frequency
Mortality	Twice a day.
Clinical Observations	Twice a day.
Local reactions	Before each administration, then 6, 24 and 48 h after each injection.
Body weight	Before each administration, daily for three consecutive days following each administration and then, at least twice a week until next administration/study end
Food consumption	At least, once during the acclimation period (4 days before the first administration) and then, twice a week, from Day 1 and throughout the study
Body temperature	Before each administration, then 6, 12, 24 h and 48 h after each dosing.
Ophthalmology	Before dosing, at the end of the main and recovery periods
Clinical pathology (including CRP)	Once before the beginning of the treatment, one and seven days after the first and last administrations, and at the end of the recovery period
Immunogenicity	Pretreatment and at the end of the main and recovery periods.
Post-mortem evaluations	3 days post last administration and end of the recovery period.

### Immunogenicity evaluation

Antibodies to the vaccine antigens were measured using a standard enzyme-linked immunosorbent assay (ELISA). ELISA plates were coated overnight with antigens. The next day, after washing and blocking, diluted control sera or test samples were added. Bound antibodies were detected using secondary antibodies. A 4-parameter logistic regression fit was used to generate a standard curve, from which antibody titers (expressed in EU/mL) were calculated. Titers were averaged from dilutions within the 20–80% segment of the standard curve. Mean titers with a coefficient of variation greater than 20% were repeated.

### Postmortem assessments

Three or 28 days after the last administration, for the main or recovery groups, respectively (as described previously^17,18^), the animals were euthanized and a necropsy was performed. Organ weights were recorded, and tissues were preserved in phosphate-buffered, neutral 4% formaldehyde, embedded in paraffin wax, sectioned at 5 µm, and stained with hematoxylin and eosin prior to examination for histopathology, following the WHO guidelines for adjuvanted vaccines^9^. Eyes and optic nerves and testis were preserved in modified Davidson’s fixative.

### Statistical analysis and data visualization

All the statistical analyses reported herein were performed on the data within the studies, not between studies. Data for each sex were analyzed separately. The normality of data distribution was assessed using either the Shapiro-Wilk test or the Kolmogorov test. If normality was not met, the data were log-transformed. If checks on log-transformed data failed, the data were rank-transformed. Homogeneity of variances between groups was tested using Levene’s test or Bartlett’s test. If these tests failed, rank transformation was applied.

For non-transformed or log-transformed data, one-way ANOVA was used to analyze absolute body weight, body weight change, clinical pathology values, terminal body weight, absolute organ weight, organ weight percentage, organ-to-body weight percentage, and organ-to-brain weight percentage. If the group effect of the ANOVA was significant (*p* ≤ 0.05), pairwise comparisons were conducted using Dunnett’s multiple comparison test or t-tests. Group comparisons were evaluated at the 0.05 and 0.01, 2-tailed probability levels. For rank-transformed data, non-parametric tests such as the Kruskal-Wallis test, Dunn test, or Wilcoxon test were used for group comparisons.

In the two earliest studies, ANCOVA was used with pretreatment data as covariates for posttreatment body weight, body temperature, and clinical pathology data. The suitability of the covariate was checked based on criteria such as sufficient cases, non-zero variability, and parallel covariate effects across groups (significance level for parallelism test 0.01). If any check for covariates, normality, or homoscedasticity failed, the checks were repeated using log-transformed data. If the checks on log-transformed, covariate-adjusted data failed, the covariate was removed, and the normality and homogeneity checks were repeated. If these checks passed on non-transformed or log-transformed data, the data was analyzed without the covariate. If they failed, the data were rank-transformed, and the covariate was reinstated.

Group mean equality was then tested by ANCOVA or ANOVA if the covariate was removed. If the group means were non-homogeneous (*p* < 0.05), pairwise comparisons of groups with the control group were conducted using Dunnett’s multiple comparison test (significance levels 0.01 and 0.05). Incidences of histopathological changes were analyzed using Fisher’s exact probability test.

The graphs were created using the R Language and Environment for Statistical Computing^19^ with the Tidyverse package^20^.

## Supplementary information

Supplementary Information_Destexhe.

## Data Availability

All data analyzed for the current manuscript were extracted from the reports of proprietary Good Laboratory Practice (GLP) for Nonclinical Safety Studies. The tables and graphs in this published article include all the data.
