# Supplementary material for: A platform approach as a plausible option for nonclinical safety assessment of adjuvanted vaccines
Source: NPJ Vaccines. 2025 Aug 13;10:192. doi: 10.1038/s41541-025-01245-3 (PMC12350924; doi:10.1038/s41541-025-01245-3)
Supplement: Supplementary file 1 — Supplementary Information_Destexhe. [file 41541_2025_1245_MOESM1_ESM.pdf]

SUPPLEMENTARY INFORMATION

Supplementary Table 1. Toxicological tests

Category	Test*
BIOCHEMISTRY	Alanine Aminotransferase
BIOCHEMISTRY	Albumin
BIOCHEMISTRY	Albumin/Globulin
BIOCHEMISTRY	Alkaline Phosphatase
BIOCHEMISTRY	Aspartate Aminotransferase
BIOCHEMISTRY	Bilirubin
BIOCHEMISTRY	Calcium
BIOCHEMISTRY	Chloride
BIOCHEMISTRY	Cholesterol
BIOCHEMISTRY	Creatine Kinase
BIOCHEMISTRY	Creatinine
BIOCHEMISTRY	Gamma Glutamyl Transferase
BIOCHEMISTRY	Glucose
BIOCHEMISTRY	Lactate Dehydrogenase
BIOCHEMISTRY	Phosphate
BIOCHEMISTRY	Phospholipid
BIOCHEMISTRY	Potassium
BIOCHEMISTRY	Protein
BIOCHEMISTRY	Sodium
BIOCHEMISTRY	Triglycerides
BIOCHEMISTRY	Urea Nitrogen
Body Temperature - Rectal	Temperature
CLINICAL CHEMISTRY	Alanine Aminotransferase
CLINICAL CHEMISTRY	Albumin
CLINICAL CHEMISTRY	Albumin/Globulin
CLINICAL CHEMISTRY	Alkaline Phosphatase
CLINICAL CHEMISTRY	Aspartate Aminotransferase
CLINICAL CHEMISTRY	Bilirubin
CLINICAL CHEMISTRY	C Reactive Protein
CLINICAL CHEMISTRY	Calcium
CLINICAL CHEMISTRY	Chloride
CLINICAL CHEMISTRY	Cholesterol
CLINICAL CHEMISTRY	Creatine Kinase
CLINICAL CHEMISTRY	Creatinine
CLINICAL CHEMISTRY	Gamma Glutamyl Transferase
CLINICAL CHEMISTRY	Globulin
CLINICAL CHEMISTRY	Glucose
CLINICAL CHEMISTRY	Lactate Dehydrogenase
CLINICAL CHEMISTRY	Phosphate
CLINICAL CHEMISTRY	Phospholipid
CLINICAL CHEMISTRY	Potassium
CLINICAL CHEMISTRY	Protein
CLINICAL CHEMISTRY	Sodium
CLINICAL CHEMISTRY	Triglycerides
CLINICAL CHEMISTRY	Urea Nitrogen
CLINICAL SIGNS	Clinical Observation
CLINICAL SIGNS	Clinical Observations
COAGULATION	Activated Partial Thromboplastin Time
COAGULATION	Fibrinogen
COAGULATION	Prothrombin Time
HEMATOLOGY	Basophils
HEMATOLOGY	Blasts
HEMATOLOGY	Blasts/Leukocytes

Category	Test*
HEMATOLOGY	Eosinophils
HEMATOLOGY	Ery. Mean Corpuscular HGB Concentration
HEMATOLOGY	Ery. Mean Corpuscular Hemoglobin
HEMATOLOGY	Ery. Mean Corpuscular Volume
HEMATOLOGY	Erythrocytes
HEMATOLOGY	Erythrocytes Distribution Width
HEMATOLOGY	Hematocrit
HEMATOLOGY	Hemoglobin
HEMATOLOGY	Large Unstained Cells
HEMATOLOGY	Leukocytes
HEMATOLOGY	Lymphocytes
HEMATOLOGY	Metamyelocytes
HEMATOLOGY	Metamyelocytes/Leukocytes
HEMATOLOGY	Monocytes
HEMATOLOGY	Neutrophils
HEMATOLOGY	Neutrophils Band Form
HEMATOLOGY	Neutrophils Band Form/Leukocytes
HEMATOLOGY	Platelets
HEMATOLOGY	Promyelocytes
HEMATOLOGY	Promyelocytes/Leukocytes
HEMATOLOGY	Reticulocytes
IMMUNOLOGY	C Reactive Protein
OPHTHALMOLOGY	Ophthalmic Observations - Indirect
OPHTHALMOLOGY	Ophthalmic Observations - Slitlamp
OPHTHALMOLOGY	Ophthalmology
PHYSICAL EXAM	Vet Assessment
	Body Weight
	Body Weight Gain
	Clinical Signs Follow-up
	Food Consumption
	General Histopathologic Exam, Qual
	Gross Pathological Examination
	Microscopic Examination
	Organ to Body Weight Ratio
	Organ to Brain Weight Ratio
	Terminal Body Weight
	Tumor Examination
	Weight

* Studies 1–3.

Supplementary Table 2. Tissues for gross pathological and/or microscopic examination and weighing.

Tissues for gross pathological and/or microscopic examination and weighing†	
ARTERY, AORTA	LIVER
BONE MARROW	LUNG
BONE MARROW, FEMUR	LYMPH NODE, ILIAC*
BONE MARROW, STERNUM	LYMPH NODE, INGUINAL
BONE, FEMUR	LYMPH NODE, MANDIBULAR
BONE, STERNUM	LYMPH NODE, MESENTERIC
BRAIN	LYMPH NODE, POPLITEAL
CERVIX	MUSCLE, BICEPS FEMORIS
EPIDIDYMIS*	MUSCLE, DIAPHRAGM
ESOPHAGUS	MUSCLE, SKELETAL
EYE	NERVE, OPTIC
GALLBLADDER	NERVE, SCIATIC
GLAND, ADRENAL	OVARY
GLAND, HARDERIAN	OVIDUCT
GLAND, LACRIMAL	PANCREAS
GLAND, MAMMARY	SITE, INJECTION
GLAND, PARATHYROID	SKIN/SUBCUTIS
GLAND, PITUITARY*	SMALL INTESTINE, DUODENUM
GLAND, PROSTATE	SMALL INTESTINE, ILEUM
GLAND, PROSTATE/GLAND, SEMINAL VESICLE	SMALL INTESTINE, JEJUNUM
GLAND, SALIVARY, PAROTID	SPINAL CORD
GLAND, SALIVARY, SUBLINGUAL	SPLEEN*
GLAND, SALIVARY, SUBMANDIBULAR	STOMACH
GLAND, SEMINAL VESICLE	TESTIS
GLAND, THYROID*	THYMUS
GLAND, THYROID/GLAND, PARATHYROID*	TONGUE
GUT-ASSOCIATED LYMPHOID TISSUE	TRACHEA
HEART	URETER
JOINT	URETHRA
KIDNEY	URINARY BLADDER
LARGE INTESTINE, CECUM	UTERUS
LARGE INTESTINE, COLON	UTERUS/CERVIX*
LARGE INTESTINE, RECTUM	VAGINA
LARYNX	WHOLE ANIMAL

† Studies 1–3

* Weighed tissues.

Supplementary Table 3. Summary of additional clinical pathology changes (hematology, clinical chemistry and coagulation) following administration of AS01-adjuvanted vaccines to rabbits

Date of Study	2019	2018	2017	2012	2012
Vaccine	Ag-A2	Ag-A1	Ag-B	Ag-C	Ag-D
Expression System	CHO	CHO	CHO	*E. coli*	CHO
**Activated Partial Thromboplastin Time**				x*	x
**Albumin**	x				
**Eosinophils**		x		x	
**Ery. Mean Corpuscular Volume**				x	
**Prothrombin Time**				x	x

* $p < 0.05$ in females
